# Dengue Burden and Factors Influencing Severity in Honduras: A Descriptive and Analytical Study

**DOI:** 10.1590/0037-8682-0594-2023

**Published:** 2024-06-10

**Authors:** Melba Zúniga-Gutiérrez, Marlon Meléndez, Saroni Shadai Rodríguez Montoya, Héctor Alcides Figueroa Escobar, Jorge López, Sandra Paz, Dilcia Sauceda-Acosta

**Affiliations:** 1 Universidad Nacional Autónoma de Honduras, Research Institute in Medical Sciences and Right to Health, Epidemiology Postgraduate Program Tegucigalpa, Honduras.; 2 Universidad Nacional Autónoma de Honduras, Epidemiology Postgraduate Program. Tegucigalpa, Honduras.; 3 Universidad Nacional Autónoma de Honduras, University Demographic Observatory, Tegucigalpa, Honduras.; 4 Universidad Nacional Autónoma de Honduras, Demography and Development Postgraduate Program. Tegucigalpa, Honduras.; 5 Ministry of Health, Health Surveillance Unit, National Virology Laboratory. Tegucigalpa, Honduras.

**Keywords:** Dengue, Arbovirus, Epidemiology, Severe dengue

## Abstract

**Background:**

Dengue is a disease that accounts for a major morbidity and mortality in Honduras.

**Methods:**

This descriptive study used an analytical component based on the data from the National Virology Laboratory between 2016-2022. Ordinal logistic regression was used to identify the factors associated with the classification of dengue without warning signs (DWOS), dengue with warning signs (DWS), and severe dengue (SD).

**Results:**

Overall, 14,687 dengue cases were included; 50.1% had DWOS, 36.5% had DWS, and 13.4% had SD. Patients that were more associated with a higher probability of DWS and SD were patients in the age groups 1-4 years (DWS OR 1.61; 95%CI:1.33-1.94), (SD OR 1.52; 95% CI:1.26-1.84), 5-9 years (DWS OR 2.01; 95% CI:1.68-2.40), (SD OR 2.00; 95% CI:1.67-2.40), and 10-19 years (DWS OR 1.55; 95% CI:1.30-1.85) (SD OR 1.57; 95% CI:1.31-1.88). The departments that were associated with a higher probability of DWS and SD were La Paz (OR 6.35; 95% CI:3.53-11.42), (OR 10.94; 95% CI:5.96-20.08), Copán (OR 6.94; 95% CI:5.05-9.53) (OR 7.33; 95% CI: 5.35-10.03), Valle (OR 5.22; 95% CI:1.25-21.82) (OR 10.71; 95% CI:2.21-51.88).

**Conclusions:**

During the study period, dengue presented endemic behavior, with peaks consistent with the last two epidemics in Honduras in 2015 and 2019. The main factors associated with dengue severity were age< 19 years, male sex, and being from La Paz, Copán, or Valle.

## INTRODUCTION

Dengue is an arbovirosis caused by a *flavivirus* (DENV), which comprises four genetically and antigenically related viruses, known as serotypes 1-4^1^. The incidence of dengue has significantly increased worldwide in the recent decades, with approximately half of the population being at risk of acquiring this infection. It is estimated that between 100 and 400 million cases occur globally each year^2^. This increase in incidence is due to factors, such as population growth, urbanization, tourism, global warming, forced displacement, barriers to preventive care, and geographic conditions that delay government interventions^3^ ^,^ ^4^. 

The epidemiology of this disease has shown essential changes, with a significant number of cases and hospitalizations in the recent years and epidemics of greater magnitude^5^. Some regions are especially vulnerable due to their geographic location^4^. The Americas have experienced substantially increased dengue morbidity and mortality in the recent decades; 2019 represented the year with the highest number of reported cases in history, with 3.1 million cases and more than 25,000 classified as severe^6^. Dengue in Central America is cyclical and endemic yearly, with epidemics occurring every three to five years^7^. Central America represents only 7.7% of the population of the Americas. However, of the 44 million inhabitants in the region, it is estimated that 10% of the population is affected by dengue, with Guatemala being the most affected country, followed by Honduras^8^.

In Honduras, the behavior of dengue is similar to that described in nearby countries, such as Guatemala and El Salvador^9^ ^,^ ^10^, with endemic or epidemic viral circulation at the national level, predominantly in the Metropolitan Health Regions of Tegucigalpa and San Pedro Sula, with the most affected population being children under 19 years of age^11^ ^,^ ^12^. Dengue continues to be responsible for a high burden of morbidity and mortality in Honduras; notably, between the years 2016 and 2023, there were 247,140 cases reported in the Honduras^13^. The incidence rate during this period ranged from 56.31 (2016) to 1,442.92 (2019)^13^. Despite being a growing public health problem, the last description of the epidemiological situation of dengue in Honduras was made more than ten years ago^11^, which is why this study aimed to provide an update on the clinical and epidemiological behavior of dengue in Honduras between 2016-2022.

## METHODS

### ● Study design

An observational, descriptive study with an analytical component was conducted on the clinical characteristics of suspected dengue patients who tested positive using real-time polymerase chain reaction (RT-PCR). 

### ● National surveillance and data collection procedures

Honduras is geographically divided into 18 departments: the largest metropolitan areas are located in the central (Francisco Morazán), northwestern (Cortés), and northern (Atlántida) zones. Dengue is a notifiable disease in Honduras, and suspected clinical cases have been detected in health units across 18 departments. The Health Surveillance Unit (HSU) of the Ministry of Health conducts epidemiological surveillance of dengue. A*suspected clinical case*of dengue is defined as a febrile illness of abrupt onset, lasting up to seven days, with two or more of the following manifestations: headache, myalgia, arthralgia, retro-ocular pain, skin rash, leukopenia, and the presence or absence of bleeding^14^.

To confirm infection with the dengue virus, the detection of genomic sequences by RT-PCR was performed in a blood sample taken within the first five days from the onset of fever or detection of immunoglobulin M (IgM) antibodies in a sample taken on the sixth day from the onset of fever. The sample was sent to the National Virology Laboratory (NVL) with an epidemiological record for arbovirus surveillance, including general patient information and demographic and clinical data. The dengue databases of the NVL of the Ministry of Health of Honduras between January 1, 2016, and June 30, 2022, were combined in a Microsoft Excel file. To obtain as many records as possible, missing data in the database were searched for in the epidemiological records. However, some records were also incomplete for the variables of interest. A flowchart is shown in Supplementary Figure 1.

### ● Case definition

The patients were clinically classified as having dengue without warning signs (DWOS), dengue with warning signs (DWS), or severe dengue (SD). A patient was diagnosed with DWS when one or more of the following symptoms were present: severe abdominal pain (SAP), persistent vomiting, ascites, pleural effusion, pericardial effusion, epistaxis, gingivorrhagia, hematemesis, melena, metrorrhagia, lethargy, irritability, postural hypotension, or hepatomegaly. Severe dengue (SD) was classified as one or more of the following: shock, respiratory distress, weak pulse, capillary refill >2 s, pulse pressure less than 20 mmHg, cold extremities, and neck stiffness. A patient was classified as having DWOS when none of the above-mentioned signs or symptoms were present^14^. 

### ● Statistical Analysis

STATA version 17.0 was used for the statistical analysis. For the graphic representation of dengue cases by department in each year studied, georeferencing was performed using QGIS. A statistical analysis was used to estimate the absolute frequencies and percentages for all categorical variables. The chi-square test was used to investigate the existence or absence of a relationship between the clinical classification of dengue and the explanatory variables. Cramér’s V statistic was used to estimate the magnitude of this relationship. A Kaplan-Meier analysis was performed to analyze the time elapsed between the onset of fever and contact with health services. Log-rank and Breslow tests were used to investigate the existence of statistically significant differences in the time elapsed between the onset of symptoms and contact with health services, stratified by sex and clinical classification of dengue. Crude Odds Ratios (OR) were calculated to estimate the strength of associations. In a multivariate analysis, the outcome variable (clinical classification of dengue) was measured on an ordinal scale. Notably, ordinal logistic regression was used; this type of regression considered optimizing the standard error in the presence of an ordinal dependent variable (event). To determine the explanatory variables associated with the clinical classification of dengue that should be included in the model, enter and stepwise statistical techniques were used as validation elements to select the main explanatory variables. The Hosmer-Lemeshow test was used to determine the goodness of fit of the model. The significance level was set at p<0.05. 

Before conducting the analyses, authorization was obtained from the ethics committee and institutional endorsements to access the dengue databases of the NVL of the Ministry of Health of Honduras.

## RESULTS

A total of 23,811 samples were received and processed during the study period; 7,086 samples were negative and 1,796 samples were eliminated due to having insufficient data, leaving a total of 14,687 PCR-positive dengue cases. Of these, 50.1% of patients (n=7,360) were classified as having DWOS, 36.5% of patients (n=5,359) as having DWS, and 13.4% of patients (n=1,968) as having SD. The highest number of patients were within the 10-19 years and 20-49 years age groups, followed by the 5-9 years age group; this behavior was maintained among the three clinical classifications. A statistically significant relationship was found between age and clinical classification (p<0.001); however, according to Cramér’s V test, this relationship was weak (p=0.1027).

Of the total number of positive patients, the most significant were women (54.2%), a behavior maintained among the clinical classifications. No significant relationship was observed (p=0.001, Cramér’s V = 0.0303). The departments with the most significant representation were Francisco Morazán (31.6%), Cortés (23.3%), and Atlántida (15.4%). A significant, but weakly significant relationship was found (p<0.001, Cramér’s V: 0.1446) (Table 1). 

**TABLE 1:** Sociodemographic characteristics of patients with dengue, 2016-2022.

General data	Total	Clinical Classification			p value^†^	Cramér's V
		Dengue without warning signs	Dengue with warning signs	Severe Dengue		
	n=14,687	n=7,360	n=5,359	n=1,968		
	n (%)	n (%)	n (%)	n (%)		
**Sex**						
Female	7,956 (54.2)	4,092 (55.6)	2,804 (52.3)	1,060 (55.9)	0.001*	0.0303
Male	6,731 (45.8)	3,268 (44.4)	2,555 (47.7)	908 (46.1)		
**Age**						
0 - 4	1,795 (12.2)	851 (11.6)	697 (13.1)	247 (12.6)	0.000*	0.1027
5 - 9	3,437 (23.4)	1,378 (18.7)	1,486 (27.7)	573 (29.1)		
10 - 19	4,144 (28.2)	2,017 (27.4)	1,533 (28.6)	594 (30.2)		
20 - 49	4,240 (28.9)	2,459 (33.4)	1,334 (24.9)	447 (22.7)		
50 - 59	509 (3.5)	310 (4.2)	151 (2.9)	48 (2.4)		
60 or more	562 (3.8)	345 (4.7)	158 (2.9)	59 (3.0)		
**Origin**						
Atlántida	2,266 (15.4)	1,228 (16.7)	840 (15.7)	198 (10.1)	0.000*	0.1446
Choluteca	92 (0.6)	46 (0.6)	35 (0.7)	11 (0.6)		
Colón	799 (5.4)	508 (6.9)	249 (4.6)	42 (2.1)		
Comayagua	294 (2.0)	235 (3.2)	53 (1.0)	6 (0.3)		
Copan	755 (5.1)	232 (3.1)	386 (7.2)	137 (7.0)		
Cortés	3,423 (23.3)	1,633 (22.2)	1,210 (22.6)	580 (29.5)		
El Paraíso	500 (3.4)	273 (3.7)	146 (2.7)	81 (4.1)		
Francisco Morazán	4,639 (31.6)	2,107 (28.6)	1,777 (33.2)	755 (38.4)		
Gracias a Dios	24 (0.2)	17 (0.2)	6 (0.1)	1 (0.05)		
Intibucá	116 (0.8)	62 (0.8)	48 (0.9)	6 (0.3)		
Islas de la Bahía	200 (1.4)	154 (2.1)	36 (0.7)	10 (0.5)		
La Paz	47 (0.3)	13 (0.2)	20 (0.4)	14 (0.7)		
Lempira	85 (0.6)	49 (0.7)	33 (0.6)	3 (0.2)		
Ocotepeque	2 (0.01)	2 (0.03)	-	-		
Olancho	530 (3.6)	310 (4.2)	200 (3.7)	20 (1.0)		
Santa Bárbara	514 (3.5)	241 (3.3)	199 (3.7)	74 (3.8)		
Valle	6 (0.04)	2 (0.03)	2 (0.04)	2 (0.1)		
Yoro	395 (2.7)	248 (3.4)	119 (2.2)	28 (1.4)		

†  p-value corresponding to the chi-square test. *Statistically significant at the 5% level (p<0.05).

Regarding the behavior of dengue throughout the seven years studied, when analyzing the distribution of total positive samples, an increase was observed in the first epidemiological week (EW) of 2016, followed by the 2015 epidemic, and then returned to the endemic pattern in 2017, presenting a trend of accumulation of cases in the last epidemiological weeks. After EW 25, 2018, the number of cases increased significantly until peaking one year later, at approximately EW 29, 2019. 

**FIGURE 1:**
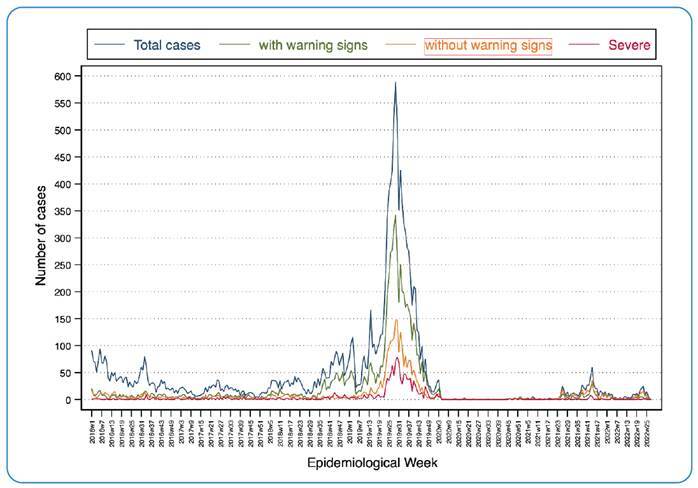
Behavior by epidemiological week according to the clinical classification of Dengue, 2016-2022.

**FIGURE 2:**
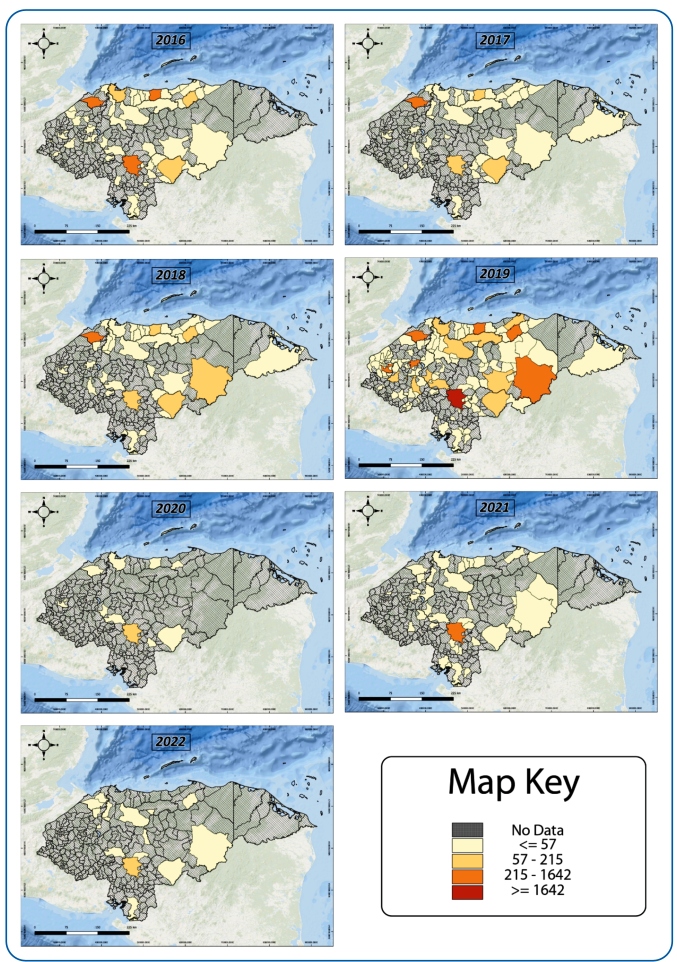
Geographical distribution: Number of cases by municipality, 2016-2022.

At this point, the number of cases began to decline until it reached its lowest levels in 2020 and the first half of 2021, a year in which positive samples begin to increase between June and July. Most cases have been observed to be concentrated in the second half of the year, which is the characteristic endemic pattern. The SD pattern remained relatively stable even during the most significant increase in the total number of dengue cases, except for the 2019 epidemic, when there was a significant increase (Figure 1). The majority of positive dengue cases were consistently located in the central zone during the study period, with an increase in cases reported in the northern zone during the seasons, and a marked increase in cases throughout the territory in 2019 (Figure 2).

The most frequent signs and symptoms according to the clinical classification of dengue are described in Table 1. Considering all the clinical classifications of dengue, the median time between the onset of symptoms and contact with health services (HS) was four days, and according to the interquartile range, [IR=2-5], 50% of patients had contact with HS between the second and fifth days. The probability of patients presenting for consultation 4 days after the onset of fever or symptoms was 0.64, 95%CI (0.63- 0.65). There was no statistically significant difference between the sexes (p=0.124). The most frequent comorbidities and other conditions among the patients are shown in Table 2. 

**TABLE 2:** Associated conditions of dengue patients according to clinical classification.

Comorbidities and medication use	Total	Clinical Classification			p-value^†^
		Dengue without warning signs	Dengue with warning signs	Severe Dengue	
	n=14,687	n=7,360	n=5,359	n=1,968	
	n(%)	n (%)	n (%)	n(%)	
Obesity	156 (1.1)	74 (1.0)	61 (1.1)	21 (1.1)	0.764
Diabetes	131 (0.9)	78 (1.1)	40 (0.7)	13 (0.7)	0.090
Arterial hypertension	130 (0.9)	83 (1.1)	38 (0.71)	9 (0.5)	0.004
NSAID ^∝^use	334 (2.3)	124 (1.7)	156 (2.9)	54 (2.7)	0.000
Anticoagulant use	6 (0.04)	4 (0.05)	2 (0.04)	-	0.865
Osteoarticular disease	3 (0.02)	2 (0.03)	1 (0.02)	-	1.000
Rheumatologic disease	6 (0.04)	2 (0.03)	4 (0.07)	-	0.357
Lupus	3 (0.02)	2 (0.03)	1 (0.02)	-	1.000
CKD^‡^	3 (0.02)	1 (0.01)	1 (0.02)	1 (0.05)	0.525
Chronic liver disease	1 (0.01)	-	1 (0.02)	-	0.499
Thrombocytopenic disease	2 (0.01)	1 (0.01)	1 (0.02)	-	1.000
Hemolytic disease	1 (0.01)	1 (0.01)	-	-	1.000
Leukemia	10 (0.07)	8 (0.11)	2 (0.04)	-	0.204
Women 15-49 years old	600 (16.8)	419 (20.0)	125 (11.6)	56 (14.3)	0.000*

^‡^ **CKD:** Chronic kidney disease ^∝^ **NSAID** ^:^ non-steroidal anti-inflammatory drugs ^†^p-value corresponding to the chi-square test. *Statistically significant at the 5% level (p<0.05).

When analyzing the time elapsed between the onset of symptoms and medical consultation, it was found that three days of IR [2-4] elapsed for patients with DWOS, four days of IR [3-5] for patients with DWS and four days of IR [3-6] for patients with SD. Likewise, when estimating the probabilities of attending a HS four days after the onset of symptoms by the clinical classification of dengue, it was observed that for DWOS, the probability was 0.81; CI95% (0.78-0.83); for DWS 0.57; CI 95% (0.56-0.58); and for SD 0.52; 95%CI (0.52-0.59). Patients classified as having DWS and SD were less likely to visit a HS within four days than those classified as having DWOS (p<0.001) (Supplementary Figure 2).

In the multivariate analysis, patients in the age groups of 1-4 years; 5-9 years and 10-19 years had a higher chance of DWS and SD. Regarding sex, males had a higher chance of developing DWS and SD than females. However, the p-value was not statistically significant for SD. The departments associated with higher probabilities of DWS and SD were La Paz, Copán, Valle, Choluteca, Santa Bárbara, Francisco Morazán, and Cortés (Table 3). The Hosmer-Lemeshow goodness-of-fit test was performed, which showed that these models were not good at explaining the event (p<0.05).

**TABLE 3:** Factors associated with dengue with alarm signs and severe dengue.

Sociodemographic characteristics	Dengue with alarm signs		Severe Dengue	
	ORc^†^; p; (CI95%)	ORaj^‡^; p; (CI95%)		ORc^†;^; p; (CI95%)
Age				
1 - 4	1.72; 0.000; (1.42, 2.07)*	1.61; 0.000; (1.33, 1.94)*	1.69; 0.000; (1.40, 2.05)*	1.52; 0.000; (1.26, 1.84)*
5 - 9	2.18; 0.000; (1.83, 2.60)*	2.01; 0.000; (1.68, 2.40)*	2.23; 0.000; (1.86, 2.66)*	2.00; 0.000; (1.67, 2.40)*
10 - 19	1.61; 0.000; (1.35, 1.93)*	1.55; 0.000; (1.30, 1.85)*	1.65; 0.000; (1.38, 1.97)*	1.57; 0.000; (1.31, 1.88)*
20 - 49	1.16; 0.094; (0.97, 1.39)	1.17; 0.092; (0.98, 1.39)	1.13; 0.173; (0.95, 1.35)	1.12; 0.204; (0.94, 1.34)
50 - 59	1.04; 0.750; (0.82, 1.32)	1.04; 0.769; (0.81, 1.32)	1.00; 0.994; (0.79, 1.27)	0.99; 0.932; (0.77, 1.26)
60 or more	Reference	Reference	Reference	Reference
Sex (male)	1.12; 0.000; (1.06, 1.19)*	1.09; 0.010; (1.02, 1.16)*	1.09; 0.003; (1.03, 1.17)*	1.06; 0.083; (1.03, 1.17)
Origin				
Atlántida	3.40; 0.000; (2.53, 4.58)*	3.54; 0.000; (2.62, 4.76)*	3.29; 0.000; (2.45, 4.42)*	3.40; 0.000; (2.53, 4.57)*
Choluteca	3.83; 0.000; (2.36, 6.23)*	4.11; 0.000; (2.52, 6.69)*	3.99; 0.000; (2.46, 6.46)*	4.26; 0.000; (2.63, 6.91)*
Colon	2.36; 0.000; (1.71, 3.24)*	2.42; 0.000; (1.76, 3.34)*	2.24; 0.000; (1.63, 3.07)*	2.28; 0.000; (1.66, 3.13)*
Comayagua	Referencia	Referencia	Referencia	Referencia
Copán	7.37; 0.000; (5.37, 10.1)*	6.94; 0.000; (5.05, 9.53)*	7.81; 0.000; (5.72, 10.68)*	7.33; 0.000; (5.35, 10.03)*
Cortés	3.84; 0.000; (2.87, 5.15)*	3.77; 0.000; (2.81, 5.05)*	4.71; 0.000; (3.52, 6.30)*	4.66; 0.000; (3.48, 6.24)*
El Paraíso	2.94; 0.000; (2.11, 4.09)*	3.16; 0.000; (2.27, 4.41)*	3.70; 0.000; (2.66, 5.16)*	4.01; 0.000; (2.87, 5.60)*
Francisco Morazán	4.26; 0.000; (3.19, 5.70)*	4.03; 0.000; (3.01, 5.40)*	4.99; 0.000; (3.73, 6.66)*	4.69; 0.000; (3.51, 6.27)*
Gracias a Dios	1.67; 0.279; (0.66, 4.19)	1.83; 0.200; (0.73, 4.64)	1.64; 0.292; (0.66, 4.08)	1.81; 0.204; (0.72, 4.55)
Intibucá	3.76; 0.000; (2.37, 5.96)*	3.95; 0.000; (2.48, 6.27)*	3.19; 0.000; (2.04, 4.98)*	3.34; 0.000; (2.13, 5.24)*
Islas de la Bahía	1.16; 0.504; (0.75, 1.79)	1.27; 0.282; (0.82, 1.97)	1.22; 0.360; (0.79, 1.89)	1.35; 0.182; (0.87, 2.08)
La Paz	6.42; 0.000; (3.58, 11.53)*	6.35; 0.000; (3.53, 11.42)*	11.11; 0.000; (6.06, 20.35)*	10.94; 0.000; (5.96, 20.08)*
Lempira	3.21; 0.000; (1.92, 5.37)*	3.71; 0.000; (2.21, 6.22)*	2.70; 0.000; (1.64, 4.46)*	3.16; 0.000; (1.91, 5.23)*
Ocotepeque	-	-	-	-
Olancho	3.07; 0.000; (2.20, 4.29)*	3.01; 0.000; (2.16, 4.21)*	2.63; 0.000; (1.90, 3.66)*	2.55; 0.000; (1.83, 3.55)*
Santa Bárbara	4.16; 0.000; (3.00, 5.79)*	4.20; 0.000; (3.02, 5.84)*	4.60; 0.000; (3.31, 6.38)*	4.61; 0.000; (3.32, 6.41)*
Valle	4.92; 0.029; (1.18, 20.51)*	5.22; 0.024; (1.25, 21.82)*	10.53; 0.003; (2.19, 50.72)*	10.71; 0.003; (2.21, 51.88)*
Yoro	2.38; 0.000; (1.67, 3.37)*	2.45; 0.000; (1.72, 3.48)*	2.37; 0.000; (1.67, 3.35)*	2.42; 0.000; (1.71, 3.44)*

† **ORc:** Odds Ratio Crude; ^‡^ **ORaj:** Adjusted Odds Ratio; (95% Confidence Intervals); *Statistically significant at 5% level (p<0.05).

## DISCUSSION

This study found that the main factors associated with dengue severity were age less than 19 years and origin from La Paz, Copán, Valle, Choluteca, Santa Bárbara, Francisco Morazán, or Cortés.

Dengue predominantly affected the young and economically active population; however, the contribution of children under 5 years of age (12.2%) and children under 10 years of age (n=3,437; 23.4%) were significant. These data contrast with those reported by Soto on the epidemiological situation of dengue in Honduras in the 1990s, who found that only 16% of dengue cases occurred in individuals under 14 years of age^15^. This increase in the total number of cases in young patients is consistent with the behavior of the virus in countries where it has circulated for more than 20 years, resulting in the accumulation of immunity in older individuals and the displacement of primary and secondary infections in younger people, resulting in a higher risk of complications^16^ ^-^ ^18^. 

During the study period, dengue presented endemic behavior, with an increase in cases coinciding with the last two epidemics in Honduras in 2015 and 2019. Dengue is observed to most frequently affect school-age children, adolescents, and young adults. Notably, dengue cases are concentrated in the large metropolitan areas of Cortés and Francisco Morazán; this behavior coincides with that reported more than a decade ago by Ávila et al. on the behavior of dengue in Honduras until 2010^11^. It is important to note that the incidence of dengue is higher in urban areas with high population densities, such as the metropolitan areas of Cortés and Francisco Morazán, which have unplanned urbanization, greater displacement of people, and problems with drinking water supply. Consequently, each year, these areas have a high incidence of the disease, eventually causing a higher probability of reinfection, which is a risk factor for disease severity. In the case of La Paz, Valle, Choluteca, and Santa Bárbara, although these departments are not densely populated areas, they have municipalities with significant socioeconomic issues and with problems of access and quality of HS that directly affect the health of the population.

The 2019 epidemic represents one of the most severe epidemics on record thus far. The trend in total cases and severity (SD cases) makes this evident. The lowest number of cases was reported immediately after the 2019 epidemic, which may be a response to the usual behavior of the virus after an epidemic; however, this may also be a result of the focus of health resources on the COVID-19 pandemic, resulting in fewer cases being detected and reported^19^.

Dengue continues to be concentrated in the large metropolitan areas of Cortés in the northwest, Francisco Morazán in the central region, and Atlántida in the north. A high population density and poor infrastructure, especially in terms of the water supply and sewerage, favor the accumulation of water in inadequate conditions within urban homes, which provide breeding sites for *Aedes aegypti* and facilitate the spread of dengue in the urban areas^20^. 

Despite the accumulation of cases in the departments of the two main cities, the patients from regions, such as Copán and La Paz were more likely to be classified as having DWS and SD. The difficulty of access to HS may contribute to this difference, as well as to the knowledge, attitudes, and practices of the inhabitants of the rural areas. The association between the rural areas and a higher risk of dengue severity or mortality has already been described in other studies; for example, a Brazilian study found that the residents of rural areas had twice the risk of mortality from SD^21^.

Sex is a factor that increases the possibility of DWS; some studies have described male sex as a predictor of the development of complications^22^. However, sex as a severity factor has not been proven in systematic reviews^23^. The median number of days from symptom onset to contact with HS was four, similar to other regional studies^24^ ^,^ ^25^. In this study, no statistically significant difference was found according to the sex of the patient; however, a statistically significant relationship was found between the severity of dengue and the possibility of attending a HS within four days. These findings are similar to those of Burattini et al., who found that attending a HS two days after the onset of fever was associated with a greater severity^26^.

The most frequent warning signs in this population were severe abdominal pain and persistent vomiting, which is consistent with the findings of other studies^27^ ^-^ ^29^. In this study, the use of NSAIDs was associated with a 1.61 more likelihood of being classified as DWS and a 1.55 more likelihood of SD. The potential risk of increased bleeding complications with NSAID use has been previously described; however, this risk has not been evaluated in clinical trials^30^. Patients aged < 19 years were more likely to have DWS or SD than those aged > 60 years. This could be partially explained by the immunity acquired by older age groups over time and the likelihood of new dengue outbreaks in the young population. However, other factors that influence the severity of dengue, such as the infectivity status of the individual and circulating serotype, should be considered in future studies^31^.

Comorbidities, such as diabetes mellitus, hypertension, and renal and liver diseases have been described as the predictors of dengue severity^23^ ^,^ ^32^. In this study, of the comorbidities present, the relationship between hypertension and clinical classification of dengue was statistically significant. The mechanisms that enable this relationship are not fully understood; however, arterial hypertension causes endothelial dysfunction and impairment of vascular regulation^33^, which could increase the likelihood of complications due to plasma leakage.

The main limitation of this study is its design, which does not allow for the evaluation of disease progression or causality. Additionally, the reliance on secondary sources of information, such as circulating serotypes, makes it impossible to recover missing data. However, the sample size mitigates this limitation.

## CONCLUSION

This study provides an update on the epidemiological and clinical behavior of dengue, a public health concern in Honduras. The pediatric age group is most affected by the complications of the disease, and urban areas continue to be the focus of dissemination. Therefore, the economic impact of dengue in Honduras needs to be explored. Continuous monitoring of the epidemic process and using this information can help evaluate the health situation for decision-making with the objective of orienting actions at the level of HS and community interventions.
