# Supplementary figures and images for: Dengue Burden and Factors Influencing Severity in Honduras: A Descriptive and Analytical Study
Source: Rev Soc Bras Med Trop. 2024 Jun 10;57:e00407-2024. doi: 10.1590/0037-8682-0594-2023 (PMC11178375; doi:10.1590/0037-8682-0594-2023)

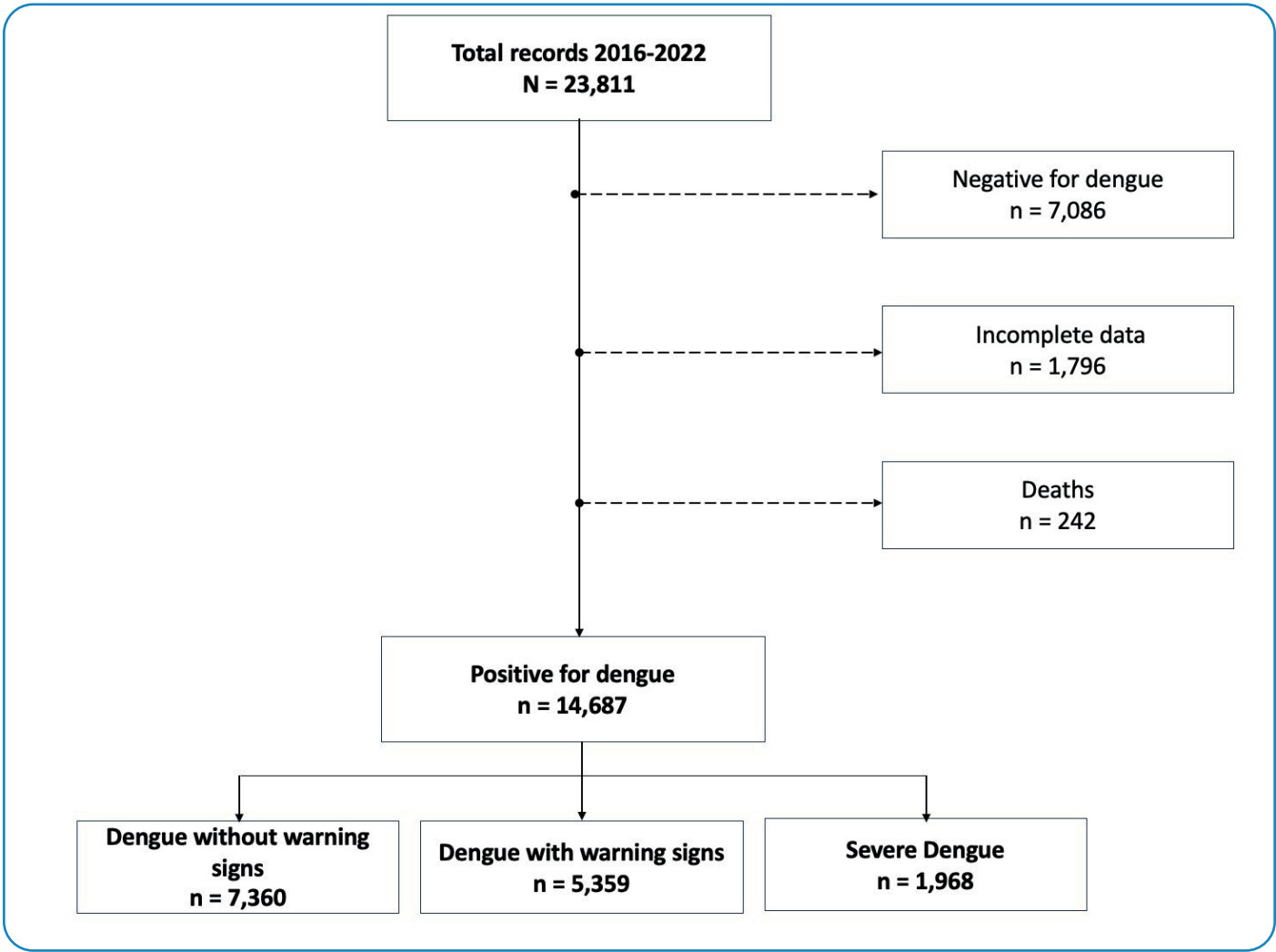

SUPPLEMENTARY FIGURE 1: Flow diagram.

Supplement: Supplementary file 1 [file 1678-9849-rsbmt-57-e00407-2024-supp1.pdf]
