# Supplementary material for: Dengue Burden and Factors Influencing Severity in Honduras: A Descriptive and Analytical Study
Source: Rev Soc Bras Med Trop. 2024 Jun 10;57:e00407-2024. doi: 10.1590/0037-8682-0594-2023 (PMC11178375; doi:10.1590/0037-8682-0594-2023)
Supplement: Supplementary file 2 [file 1678-9849-rsbmt-57-e00407-2024-supp2.pdf]

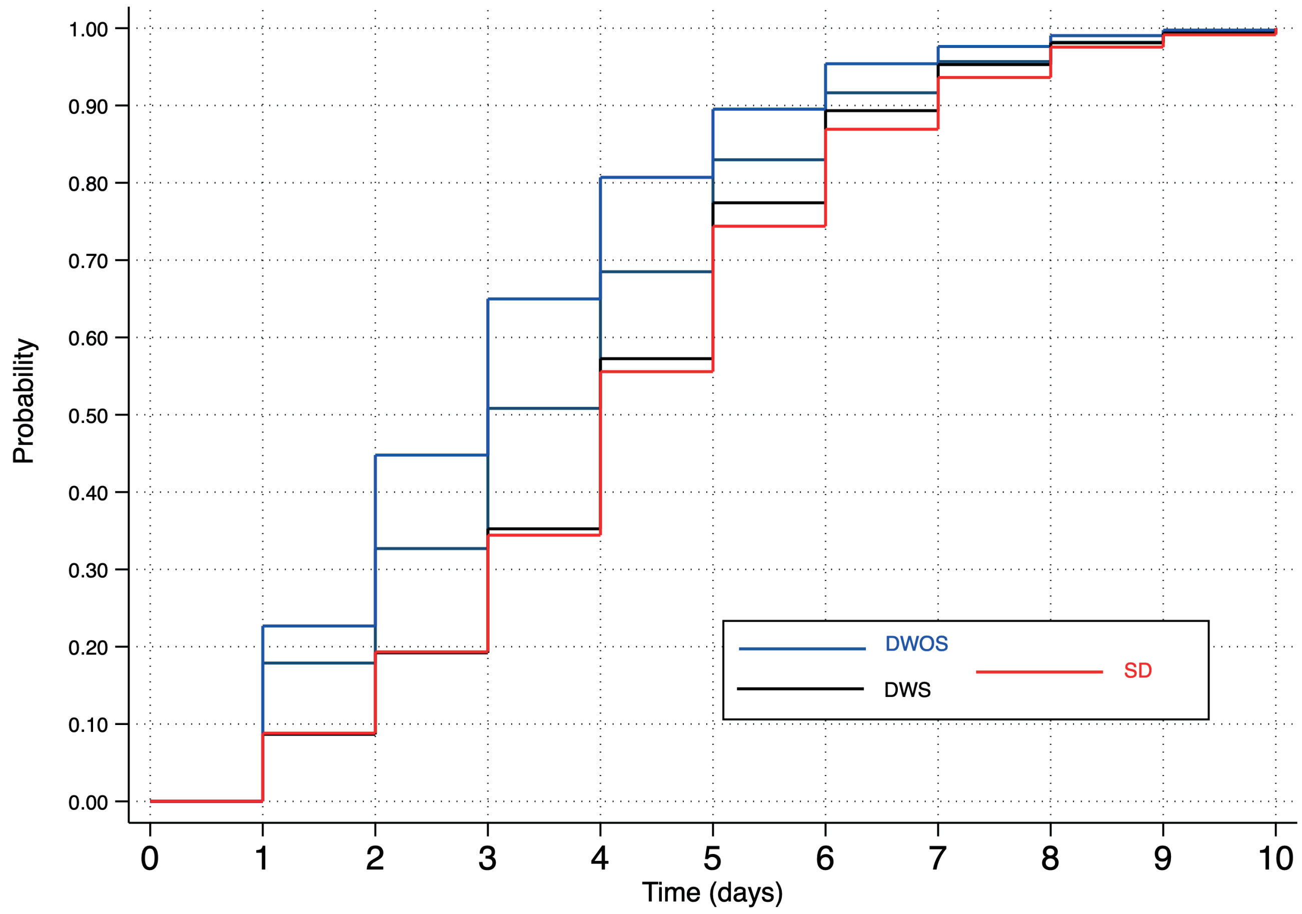

SUPPLEMENTARY FIGURE 2: Probability of attending a health service after the onset of symptoms by the clinical classification of dengue.
